# Parrot bornavirus in naturally infected Brazilian captive parrots: Challenges in viral spread control

**DOI:** 10.1371/journal.pone.0232342

**Published:** 2020-06-24

**Authors:** Aila Solimar Gonçalves Silva, Tânia Freitas Raso, Erica Azevedo Costa, Sandra Yuliet Marin Gómez, Nelson Rodrigo da Silva Martins

**Affiliations:** 1 Laboratory of Avian Diseases, Veterinary College, Universidade Federal de Minas Gerais, Belo Horizonte, Minas Gerais, Brazil; 2 Avian Ecopathology Laboratory, School of Veterinary Medicine and Animal Science, University of São Paulo, São Paulo, Brazil; 3 Animal Virology Research Laboratory, Veterinary College, Universidade Federal de Minas Gerais, Belo Horizonte, Minas Gerais, Brazil; Oklahoma State University, UNITED STATES

## Abstract

Psittaciform orthobornaviruses are currently considered to be a major threat to the psittacine bird population worldwide. Parrot bornavirus (PaBV) was identified recently in Brazil and, since then, few studies have been conducted to understand the epidemiology of PaBV in captive psittacine birds. In the present study, natural infections by PaBV in South American parrots were investigated in two breeding facilities: commercial (A) and conservationist (B). Thirty-eight psittacine of 21 different species were presented for postmortem examination. Tissue samples were collected and investigated for the presence of PaBV-RNA using RT-PCR. In addition, clinical information about these birds was used when available. PaBV infection was detected in 73.7% of all birds investigated, indicating a wide dissemination of this virus in both facilities. From birds investigated in aviary A, 66.7% showed clinical signs, 100% had typical lesions of proventricular dilatation disease (PDD), 100% had mild to severe proventricular dilatation and 88.9% were PaBV-positive. In birds from aviary B, 27.6% showed clinical signs, 65.5% had typical lesions of PDD, 62% had mild to severe proventricular dilatation and 69% were PaBV-positive. Neurological disease was observed more frequently than gastrointestinal disease. Sequencing analysis of the matrix gene fragment revealed the occurrence of genotype 4 (PaBV-4) in both places. About 15.8% of birds in this study are threatened species. We discussed the difficulties and challenges for controlling viral spread in these aviaries and implications for South American psittacine conservation. These results emphasize the urgent need to develop a national regulatory and health standard for breeding psittacine birds in the country.

## Introduction

The proventricular dilatation disease (PDD) is an emerging worldwide psittacine disease and it has become a major concern in the conservation of endangered species [1–3]. PDD was first described in the 1970s as macaw wasting disease, and then as neuropathic gastric dilatation disease, resulting in high fatality rates. Clinical signs and lesions are related to progressive gastrointestinal tract and/or neurological dysfunction, including progressive weight loss, regurgitation, undigested food in feces; crop, proventricular and intestinal stasis, proventricular dilatation, ataxia and proprioceptive deficits [4].

Avian Bornavirus (ABV), a member of family Bornaviridae, was identified as a cause of PDD in 2008 [5,6]. Bornaviruses are enveloped viruses with a non-segmented, linear, single-stranded negative sense RNA genome, previously known for causing neurologic disease in horses and sheep in Central Europe [7,8]. The International Committee on Taxonomy of Viruses (ICTV) updated the taxonomy of the order *Mononegavirales* in 2018. The previously established genus *Bornavirus* was renamed *Orthobornavirus* and, currently, there are five species in this genus affecting birds; two of them identified in psittacines (*Psittaciform 1 orthobornavirus* and *Psittaciform 2 orthobornavirus*). Psittaciform 1 orthobornavirus includes five viruses, named parrot bornavirus 1 to 4 and 7 (PaBV-1 to 4, PaBV-7), and Psittaciform 2 orthobornavirus includes only the parrot bornavirus 5 (PaBV-5) [9]. At least four additional bornaviruses remain unclassified, including PaBV-6 and PaBV-8.

The epidemiology of avian bornavirus (ABV) infections is still not well characterized [3, 5, 6]. However, studies with experimental virus inoculation using various routes of infection have been performed [10–15]. Although some of these ABV have been able to induce clinical disease and characteristic lesions, several aspects of PDD pathogenesis are still not elucidated. Even though the epidemiological studies of parrot bornavirus have increased substantially in different parts of the world in recent years, few studies have been conducted in captive psittacine in Brazil [16–18]. Nevertheless, PDD has already been described in Brazilian threatened species, such as Spix's macaw (*Cyanopsitta spixii*), extinct in the wild [19]. This raises a concern about the potential negative impact of disease in psittacine conservation.

The market of exotic birds has developed significantly in Brazil in the last decade. Currently, birds represent the second largest pet population in country [20]. This is particularly evident in psittacine birds, and consequently, trade in exotic and native species has soared. In contrast, the official regulations regarding the sanitary aspects of these kind of aviaries have not evolved, which can cause an unprecedented impact on natural parrot populations in wild.

In the present study, natural infections of avian bornavirus in psittacine birds were investigated in two institutions with different management conditions. We also discuss the difficulties and challenges for controlling viral spread in different types of facilities and the implications for trade and conservation of South American Psittaciformes.

## Material and methods

### Ethics statement

This study has Brazilian the Environmental Ministry authorization (SISBIO 54185–1) and approval of the Ethics Committee of the Universidade Federal de Minas Gerais (UFMG), Brazil (protocol 218/2016).

### Birds sampling

Psittacine carcasses were received at the university from two aviaries in the state of Minas Gerais, Brazil. One of them is commercial (A) and other one, conservationist (B). Both facilities are 51 km distance from another one and had presented previous history of suspicious cases of PDD. Between July 2015 and September 2016, dead native psittacine birds from both aviaries were received by the Avian Diseases Laboratory of the Veterinary College, UFMG and necropsy was performed in all of them.

Although several birds had not been clinical signs identified by breeders before death, all clinical information when available were obtained. Gastrointestinal signs consistent with PDD were considered, such as progressive weight loss, anorexia, crop stasis, regurgitation, inappetence, presence of undigested seeds in feces. Neurological signs considered were abnormal behavior such as increased aggression, fear and/or irritability, proprioceptive or motor deficits, tremors, ataxia, weakness, depression, seizures and sudden death.

Based on the constitution of the pectoral musculature, body condition score was determined as strongly emaciated, emaciated, moderate and good condition (0 to 3 scale). During necropsy, tissue samples from neurological central system were at first collected due to the neurotropism of ABV and to preserve sample quality. Other tissues were collected in sequence and stored at -80°C for laboratorial analyses. The main gross lesions suggestive of PDD were observed, including dilatation of proventriculus, that were scored as no dilatation (0), mild to moderate (1) or severe (2) dilatation.

### Molecular analyses

RNA was extracted from tissue samples using the guanidine thiocyanate phenol chloroform (Trizol^®^, Invitrogen, CA) based on the manufacturer's instructions. First strand cDNA was generated using the reverse transcription PCR (RT-PCR) (MMLV, Promega, USA) and specific primers of matrix (M) gene (ABVMF and ABVMR) [6] and nucleoprotein (N) gene (ABVNF1 and ABVNF2) [1]. Nested PCR was performed with primers designed in this study. This primer set (ABVNF2 and ABVNR2) was used targeting the N-gene located between nucleotide positions 632 and 1020 derived from the *Aratinga solstitialis* “bil” *Bornavirus* sequence (GeneBank accession number EU781967), the first ABV isolates [1].

Primer sequences and their respective annealing temperatures are described in Table 1. Reverse transcription was performed using 2 μg/μL of total tissue RNA and 5μM of primer ABVMF and ABVMR for the matrix (M) gene in 5μL 5X Promega buffer, (250mM Tris-HCl, 375mM KCl, 15mM MgCl_2_), 1.25μL DNTP 10mM, 1.25μL RNAsin Promega, 1μL de MMLV and 1.5μL of ultrapure DEPEC water, and incubated at 42°C for 60 minutes and stored at -20°C.

**Table 1:** Primer sequences, amplification product size, and annealing temperature (Tm) used for diagnosis of PaBV.

Primer	Sequence (5’–3’)	Product Size	Tm	Reference
PaBVMF	GGRCAAGGTAATYGTYCCTGGATGGCC	360bp	57°C	Kistler et al., 2008
PaBVMR	CCAACACCAATGTTCCGAAGMGC			
PaBVNF1	CATGAGGCTATWGATTGGATTA	389bp	50°C	Weissenböck et al., 2009
PaBVNR1	TAGCCNGCCMKTGTWGGRTTYT			
PaBVNF2 (nested)	TGAAAGCCCTGGCAAAGAGT	231bp	50°C	This article
PaBVNR2 (nested)	ATCGCGTCGGAATGACGAAT			

For amplification of the M protein gene, 10pmol of primers ABVMF and ABVMR were added to 2.5μL of 10 X PCR buffer (Phoneutria, Brazil), 0.75μL MgCl_2_ 50mM, 1μL of dNTP mix 10mM (Phoneutria, Brazil), 0.3μL of Taq DNA polymerase (Phoneutria, Brazil), 16.45μL of ultrapure DEPEC water and 2μL of cDNA. Reagent concentrations were identical in both reactions, excepting MgCl_2_ concentration, which was 3 mM in first reaction (protein M gene). For all reactions, positive and negative control were included. PCR products were revealed by electrophoresis (120V/40min) in agarose 1% stained by GelRed (Biotium, USA).

Initially, all brain samples were tested for RT-PCR which amplifies part of gene encoding matrix (M) protein. Afterwards, all negative samples were tested for RT-PCR that amplifies part of gene encoding viral nucleoprotein (N).

### Sequence analysis

PCR products were sequenced by the dideoxynucleotide method [21] in an automated capillary sequencer (ABI 3730, Applied Biosystems^®^, USA), using Big Dye Terminator^®^ Mix (Applied Biosystems, EUA) with analyses performed in version 5.2 of Applied Biosystems Sequencing Analysis. Sequencing was performed in triplicate in both directions. Sequences were compared by BLAST^®^ (https://blast.ncbi.nlm.nih.gov) and were aligned by Clustal W with sequences of matrix protein gene available in the GenBank [22]. A phylogenetic tree was constructed using the Neighbor-joining method [23] and the evolutionary distance model Kimura 2 and bootstrap with 1,000 repetitions using MEGA 7.0 software, including an external group (MH190827.1 Mammalian 1 Orthobornavirus) [24, 25].

## Results

A total of 38 birds were postmortem analyzed in this study, nine from aviary A and 29 from aviary B, representing 21 species of 10 genera. PaBV was detected in 28 birds (73.7%) representing 18 species (Table 2). Among them, there were two endangered species (EN), four vulnerable (VU), four near threatened (NT) and only eight least concerned (LC) according to the Red List of IUCN [26].

**Table 2:** Species, clinical signs, gross lesions, and PaBV detection in naturally infected psittacine birds.

Bird #	Specie of birds	Common name	Aviary	Clinical signs	Body score^#^	PDD suggestive lesions	score dilatation*	PaBV detection	IUCN status
1	*Amazona aestiva*	Blue-fronted Amazon	B	NA	0	yes	1	neg	Near Threatened
2	*Amazona brasiliensis*	Red-tailed Parrot	B	neurological	1	yes	1	pos	Near Threatened
3	*Amazona brasiliensis*	Red-tailed Parrot	B	neurological	0	yes	1	pos	Near Threatened
4	*Amazona festiva*	Southern Festive Parrot	B	NA	1	yes	1	pos	Near Threatened
5	*Amazona ochrocephala*	Yellow-crowned Parrot	A	neurological/ gastrointestinal	3	yes	2	pos	Least concern
6	*Amazona pretrei*	Red-spectacled Parrot	B	neurological	2	yes	1	neg	Vulnerable
7	*Amazona rhodocorytha*	Red-browed Parrot	B	no signs	2	no	0	pos	Vulnerable
8	*Amazona rhodocorytha*	Red-browed Parrot	B	NA	2	no	1	pos	Vulnerable
9	*Amazona rhodocorytha*	Red-browed Parrot	B	NA	1	no	1	neg	Vulnerable
10	*Amazona rhodocorytha*	Red-browed Parrot	B	NA	3	no	0	neg	Vulnerable
11	*Amazona rhodocorytha*	Red-browed Parrot	B	NA	0	no	1	neg	Vulnerable
12	*Amazona vinacea*	Vinaceous-breasted Parrot	B	neurological/ gastrointestinal	2	yes	0	pos	Endangered
13	*Amazona vinacea*	Vinaceous-breasted Parrot	B	neurological	3	yes	0	pos	Endangered
14	*Amazona vinacea*	Vinaceous-breasted Parrot	B	NA	0	yes	1	pos	Endangered
15	*Anodorhynchus hyacinthinus*	Hyacinth macaw	A	neurological	1	yes	1	pos	Vulnerable
16	*Ara ararauna*	Blue-and-yellow macaw	A	neurological	1	yes	2	pos	Least concern
17	*Aratinga jandaya*	Jandaya Parakeet	A	NA	2	yes	1	neg	Least concern
18	*Aratinga weddellii*	Dusky-headed Parakeet	A	neurological	0	yes	1	pos	Least concern
19	*Eupsittula aurea*	Peach-fronted Parakeet	B	neurological/ gastrointestinal	0	yes	1	pos	Least concern
20	*Eupsittula aurea*	Peach-fronted Parakeet	B	NA	0	yes	1	pos	Least concern
21	*Guaruba guarouba*	Golden Parakeet	B	NA	0	yes	2	pos	Vulnerable
22	*Guaruba guarouba*	Golden Parakeet	B	no signs	2	no	0	pos	Vulnerable
23	*Guaruba guarouba*	Golden Parakeet	B	no signs	2	yes	0	neg	Vulnerable
24	*Guaruba guarouba*	Golden Parakeet	B	no signs	0	no	1	neg	vulnerable
25	*Pionites leucogaster*	Green-thighed parrot	A	neurological/ gastrointestinal	1	yes	1	pos	Endangered
26	*Pionites leucogaster*	Green-thighed parrot	A	NA	1	yes	2	pos	Endangered
27	*Pionites leucogaster*	White-bellied parrot	B	NA	2	no	0	neg	Endangered
28	*Pionus fuscus*	Dusky Parrot	B	NA	2	yes	2	pos	Least concern
29	*Pionus fuscus*	Dusky Parrot	B	NA	2	no	0	pos	Least concern
30	*Pionus maximiliani*	Scaly-headed Parrot	B	NA	3	yes	2	pos	Least concern
31	*Pionus maximiliani*	Scaly-headed Parrot	B	NA	2	yes	0	neg	Least concern
32	*Pionus menstruus*	Blue-headed Parrot	B	NA	1	no	0	pos	Least concern
33	*Pionus reichenowi*	Blue-breasted parrot	A	neurological	0	yes	2	pos	Vulnerable
34	*Primolius maracana*	Blue-winged Macaw	B	NA	0	yes	2	pos	Near Threatened
35	*Primolius maracana*	Blue-winged Macaw	B	NA	1	yes	1	pos	Near Threatened
36	*Psittacara mitratus*	Mitred parakeet	A	NA	0	yes	2	pos	Least concern
37	*Triclaria malachitacea*	Blue-bellied Parrot	B	neurological	0	yes	2	pos	Near Threatened
38	*Triclaria malachitacea*	Blue-bellied Parrot	B	neurological	2	yes	0	pos	Near Threatened

^#^Body condition score: 0 (strongly emaciated), 1 (emaciated), 2 (moderate condition) and 3 (good condition)

*Score dilatation of proventriculus: 0 (no dilatation), 1 (mild to moderate), 2 (severe dilatation).

NA: information not available

Neg = negative. Pos = positive

Fourteen (36.8%) birds had clinical signs compatible with PDD (66.7% in aviary A and 27.6% in aviary B). Neurologic signs were presented by ten of them and four birds showed both gastrointestinal and neurological signs. The most common signs were emaciation, inappetence, regurgitation or presence of undigested seeds in feces, abnormal behavior (irritability), ataxia, tremors, and occasionally, seizures. Unfortunately, there was no information available on the clinical history of 20 birds (52.6%). However, in 15 of these cases, proventricular dilatation (mild to severe) was observed at necropsy and 13 cases of those were PaBV-positive.

**Fig 1:**
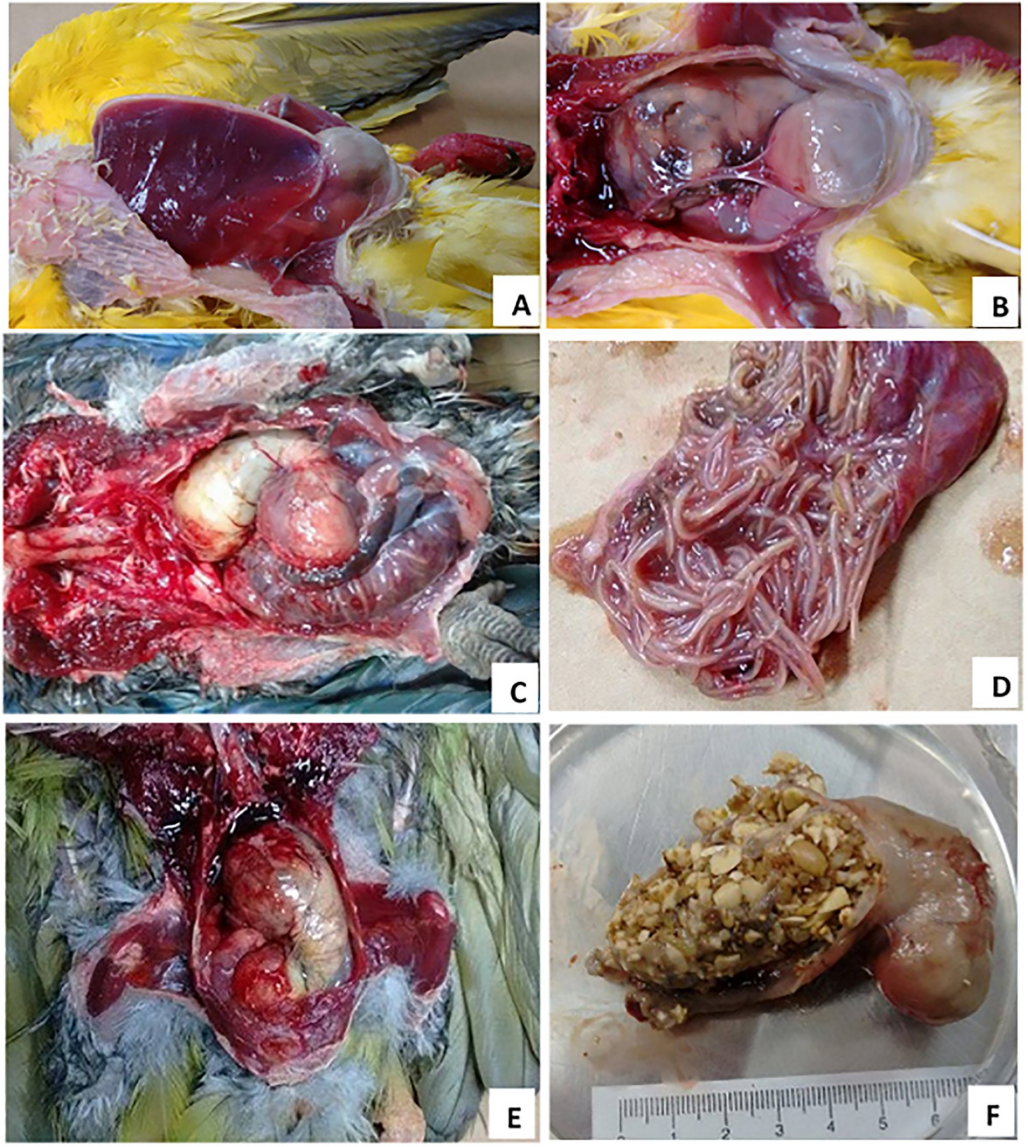
(A) Necropsy of bird #21 showing severe atrophy of pectoral muscles and a prominent ventriculus in the abdomen. (B) Bird #21 showing dilated and flaccid proventriculus and ventriculus. (C) Necropsy photo of a bird #28 showing the displacement of organs caused by dilated proventriculus and ventriculus. Note that the small intestine is also dilatated by the presence of parasites. (D) Numerous ascarid parasites in the lumen of small intestine of bird #28. (E) Necropsy photo of bird #34 showing proventriculus markedly dilated and through the thin wall it is possible to observe large accumulation of undigested food in the lumen. (F) Proventriculus dilated and filled of undigested food.

In aviary A (commercial), clinical signs and gross lesions suggestive of PDD were present in 66.7% and 100% of birds, respectively. In aviary B (conservationist), 27.6% of birds had clinical signs and 65.5% had lesions suggestive of PDD (Fig 1). PaBV was detected in 88.9% (8/9) of birds in aviary A and in 69% (20/29) of birds in aviary B. Concomitant diseases were observed in two birds, a parasitic infestation (Fig 1D) and a candidiasis.

**Fig 2:**
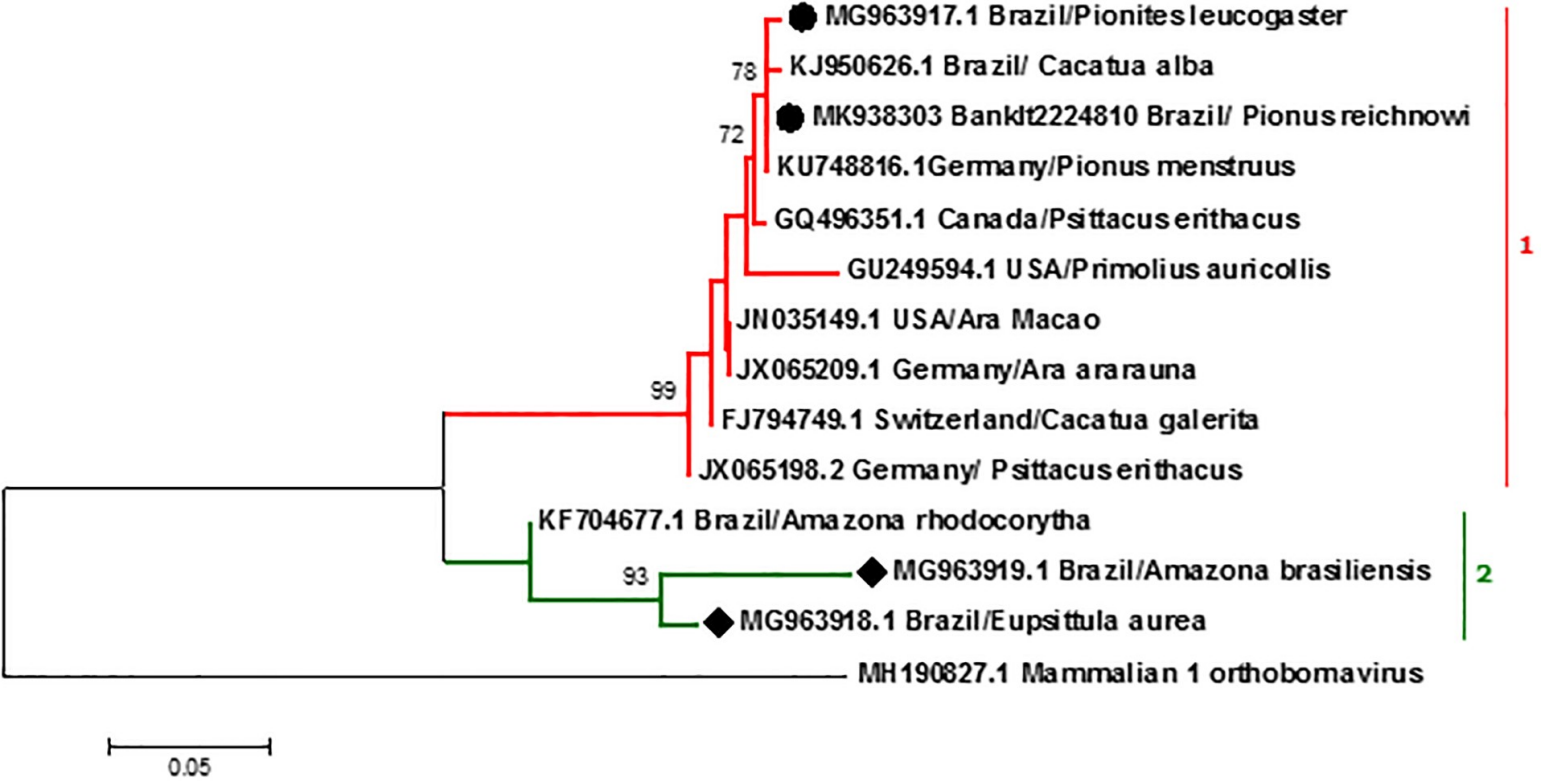
Maximum likelihood phylogenetic tree constructed from partial M gene sequence of encoding PaBV4. The variants are identified by Genbank number, parrot species and country. Sequences were analyzed using the Neighbor-Joining tree build method and the evolutionary distance model Kimura 2. Bootstrap values 1,000 are shown next to the branches. Evolutionary analyzes were performed in MEGA 7, involving 13 nucleotide sequences represented in S1 Table. Sequences identified in this study are emphasized in aviary A (●) and aviary B (◆).

Four PCR products encoding protein M were sequenced, bird #33 (MK938303.1) and #25 (MG963917.1) from aviary A and bird #20 (MG963918.1) and #3 (MG963919.1) from aviary B. The phylogenetic analysis was performed by comparing Psittaciform orthobornavirus sequences recovered in this study with other sequences available in Genbank, detailed information is provided as Supporting Information (S1 Table). The choice of sequences was based on standard viral sequences that characterizes the Psittaciform orthobornavirus according to ICTV (Fig 2). All sequences identified in this study were classified as PaBV-4. In aviary A, bird #33 (*Pionus reichnowi*) presented the highest identity sequence (99.7%) with a sequence from Germany (KU748816.1 *Pionus menstruus*). Meanwhile, the sequence from bird #25 (*Pionites leucogaster*) presented a high identity (99.0%) and nucleotide similarity with the sequence from an exotic bird in Canada (GQ 496351.1 *Psittacus erithacus*) and high identity (99.30%) with the sequence from an exotic bird imported from North America to São Paulo, Brazil (KJ950626.1 *Cacatua alba*). The identity between #33 and #25 sequences was 97.4%. In aviary B, sequences #20 (*Eupsittula aurea*) and #3 (*Amazona brasiliensis*) presented higher degrees of identity, 94.8% and 92.8% respectively, with Brazilian sequence BH1 (KF704677.1 *Amazona rhodocorytha*). The identity between #20 and #3 sequences was 93.7%.

## Discussion

Since the discovery of PaBV in 2008, several studies have been carried out demonstrating PaBV-4 as a predominant genotype in Europe and North America [1, 3, 11, 27, 28]. In Brazil, PaBV was first identified in 2014 and since then we have only identified PaBV-4 [16, 17] and the unclassified PaBV-8 [17]. Supporting this observation our results corroborate it once we demonstrate a high prevalence (73.7%) of PaBV-4 in these two Brazilian psittacine aviaries.

A total of 14 (36.8%) clinical cases were suggestive of PDD and 13 of these were PaBV-positive (Table 1). Neurological signs were observed more frequently than gastrointestinal signs, probably due to the greater evidence of the clinical manifestation. In 52.6% (20/38) of cases no information was available (NA) about the clinical situation of the birds suggesting that birds were not being frequently observed by those responsible persons. In this group, the mild to severe proventricular dilatation was observed (n = 15) and PaBV-positive birds (n = 13) were detected. This confirms that the constant lack of surveillance of the birds can seriously compromise the precision of diagnosis.

Four birds (10.5%) had no clinical signs and three of them had no gross lesions at necropsy. However, two were PaBV positive. Infected psittacines may not show clinical disease for a few weeks or even months, due to the chronic nature of the infection, and its association with an inflammatory disease. Infected asymptomatic individuals are considered essential for the dissemination and can demonstrate a high transmission rate of the virus [29, 30].

Concerning the body condition score, 57.9% (22/38) of the birds were strongly emaciated or emaciated (score 0–1), eighteen of them (81.2%) positive for PaBV. In addition, 20 of these birds had proventricular dilatation with a moderate (1) to severe (2) score. This data indicates a strong relationship of body condition score and proventricular dilatation in postmortem findings and the presence of the virus in these birds.

A history of suspected PDD cases in aviary A was reported in 2013, with low mortality occurring in young macaws but PaBV diagnosis was made only in a bird. No information was available from previous years. The collection in aviary A consist of about 1,000 specimens, composed of 30 native species and 20 exotic bird species from different origins and ages. The husbandry in aviary A was characterized by intense commerce of species such as importation and exportation activities for other countries with no adequate quarantine and national trade. As an example, the introduction of a imported group of 200 exotic *Psittacula krameri* was carried out without any health criteria. Also, if a bird became ill, it was placed in an infirmary for observation or supportive treatment by the local veterinarian, without control measures. Human visitation is allowed in this facility.

In aviary B, the collection was exclusively of native psittacine birds (about 400 birds of 34 species) and no visitors were allowed. Specimens were routinely integrated to the resident flocks from the rehabilitation centers, including from other states without adequate quarantine, as reported for aviary A. Due to small staff and absence of a frequent veterinarian, in about half of cases there was no information available concerning birds or their clinical signs. Occasionally, when a sick bird was identified, it was housed in a veterinary room for observation or supportive treatment, even without the presence of a veterinarian. Birds were relocated frequently to a new cage or group, resulting in constant stress. The first PDD outbreak reported in Brazil was described in this aviary between 2010–2011. A reemergence of cases has happened in 2013 affecting 20 birds, resulting in four out of ten birds evaluated were positive for PaBV [16]. Despite the fact there was no introduction of new birds in this aviary since 2012, no other sanitary or biosecurity measures have been instituted at this place.

Although there was a sequence identity and phylogenetic clustering with sequences of PaBV-4, differences were observed between sequences from each aviary (Fig 2). When compared to sequences previously deposited in GenBank, the sequences from aviary A showed greater similarity to sequences from other countries [3, 31, 32] and one Brazilian sequence [17] (clade 1). Aviary B sequences (clade 2) were similar to sequence previously described in a bird from this same site [16]. This was expected since no new birds were introduced into this collection since the first case of PDD was confirmed.

Our findings, therefore, suggest that in aviary A there is a viral circulation more evident with sequences from other countries. Regarding the similarity to Brazilian sequence, this originated from an exotic bird (*Cacatua alba*) imported from North America to Brazil that had traveled between these countries at least twice [17]. This diversity of sequences in aviary A may be due to several management factors, mainly the high number of imported birds, breeding of mixed species, and constant transit of birds in this facility.

Moreover, our results show differences between aviary A and B sequences, although of the same genotype, which may be responsible for the variations in the behavior of ABV infection. Studies indicate that there are differences between strains of same genotype, resulting in differences in host immune response and virulence, with PaBV-4 being suggested as the most pathogenic [14]. However, further studies associating clinical, immune and molecular aspects are still needed to better understand this process.

Several factors may have contributed to the emergence and maintenance of PaBV in both aviaries, besides the difficulty to control viral spread. Failures in the biosecurity and quarantine strategies were the most evident. Biosecurity strategies for captive collections must consider the existence of subclinical infections, since birds may remain infected without signs, at least for a few weeks, enabling transmission and high epidemiological risk [29, 30]. The large percentage of sub clinically infected birds indicates the potentially high risk of new untested birds into premises, also enabling coinfections by different ABV genotypes. Nevertheless, the resistance in accepting the definitive diagnosis of PaBV by breeders, especially in asymptomatic or mild cases, is an additional difficulty. Once there are no requirements for veterinarians to report the occurrence of bornavirus and there are no standards about diagnosis and control of this disease in the country, breeders do not agree to establish adequate control measurements. Additionally, some veterinarians fail to provide advice to breeders regarding proper quarantine and routine biosafety rules.

Beyond that, other factors contribute to this scenario, such as the lack of technical staff training, absence of a constant veterinarian on site, daily non observation of birds and no isolation of infected birds, as well as usual relocation of birds and refusal to use euthanasia as a control measure.

Finally, the presence of PaBV-4 in important breeders in Brazil reveals an epidemiological connectivity among North America, Canada and Germany PaBV sequences. The impact of ABV infections in captive native and exotic psittacine birds is growing in Brazil, especially regarding threatened species, indicating that a real risk for wild populations must be considered. A better understanding of PaBV in Brazil is crucial in the prevention of virus dissemination, the genotypes exchanges and to minimize the potential impacts to native bird species health.

In summary, this study shows the urgent need to develop national specific regulatory and health requirement for the commerce of Psittaciformes in Brazil. In this context, additional studies on epidemiology and control of PaBV in Brazil may help support the development of official sanitary programs for psittacine birds, mitigating viral spread in national and international trade and its consequent impact on biodiversity conservation.

## Supporting information

S1 Table References of the sequences used in the phylogenetic analysis. (DOCX)
