# Supplementary material for: Parrot bornavirus in naturally infected Brazilian captive parrots: Challenges in viral spread control
Source: PLoS One. 2020 Jun 24;15(6):e0232342. doi: 10.1371/journal.pone.0232342 (PMC7313744; doi:10.1371/journal.pone.0232342)
Supplement: S1 Table — (DOCX) [file pone.0232342.s001.docx]

**Table S1. References of the sequences used in the phylogenetic analysis.**

| **Genbank** | **Reference** |
| --- | --- |
| KF704677.1 | 1 |
| GU249594.1 | 2 |
| MH190827.1 | 3 |
| JN035149.1 | 4 |
| KJ950626.1 | 5 |
| GQ496351.1 | 6 |
| JX065198.2 | 7 |
| JX065209.1 | 7 |
| KU748816.1 | 8 |
| FJ794749.1 | 9 |
| MK938303.1 | This study |
| MG963917.1 | This study |
| MG963918.1 | This study |
| MG963919.1 | This study |

1. [Donatti RV](https://www.ncbi.nlm.nih.gov/pubmed/?term=Donatti%20RV%5BAuthor%5D&cauthor=true&cauthor_uid=24758135), [Resende M](https://www.ncbi.nlm.nih.gov/pubmed/?term=Resende%20M%5BAuthor%5D&cauthor=true&cauthor_uid=24758135), [Ferreira FC](https://www.ncbi.nlm.nih.gov/pubmed/?term=Ferreira%20FC%5BAuthor%5D&cauthor=true&cauthor_uid=24758135), [Marques MV](https://www.ncbi.nlm.nih.gov/pubmed/?term=Marques%20MV%5BAuthor%5D&cauthor=true&cauthor_uid=24758135), [Ecco R](https://www.ncbi.nlm.nih.gov/pubmed/?term=Ecco%20R%5BAuthor%5D&cauthor=true&cauthor_uid=24758135), [Shivaprasad HL](https://www.ncbi.nlm.nih.gov/pubmed/?term=Shivaprasad%20HL%5BAuthor%5D&cauthor=true&cauthor_uid=24758135), [de Resende JS](https://www.ncbi.nlm.nih.gov/pubmed/?term=de%20Resende%20JS%5BAuthor%5D&cauthor=true&cauthor_uid=24758135), [Martins NR](https://www.ncbi.nlm.nih.gov/pubmed/?term=Martins%20NR%5BAuthor%5D&cauthor=true&cauthor_uid=24758135). Fatal proventricular dilatation disease in captive native psittacines in Brazil Avian Dis. 2014; 58 (1): 187-193. doi: [10.1637/10588-061013-Case.1](https://doi.org/10.1637/10588-061013-Case.1)
2. Gray P, Hoppes S, Suchodolski P, Mirhosseini N, Payne S, Villanueva I, et al. Use of avian bornavirus isolates to induce proventricular dilatation disease in conures. Emerg Infect Dis. 2010;16(3):473–9. PMID:20202423. doi:10.3201/eid1603.091257
3. Korn K, Coras R, Bobinger T, [Herzog](https://pubmed.ncbi.nlm.nih.gov/?term=Herzog+SM&cauthor_id=30281979) S M,  [Lücking](https://pubmed.ncbi.nlm.nih.gov/?term=L%C3%BCcking+H&cauthor_id=30281979) H, [Stöhr](https://pubmed.ncbi.nlm.nih.gov/?term=St%C3%B6hr+R&cauthor_id=30281979) R, [Huttner](https://pubmed.ncbi.nlm.nih.gov/?term=Huttner+HB&cauthor_id=30281979) H B, [Arndt Hartmann](https://pubmed.ncbi.nlm.nih.gov/?term=Hartmann+A&cauthor_id=30281979) A, [Ensser](https://pubmed.ncbi.nlm.nih.gov/?term=Ensser+A&cauthor_id=30281979) A. Fatal Encephalitis Associated with Borna Disease Virus 1. N Engl J Med. 2018;379(14):1375‐1377. doi:10.1056/NEJMc1800724
4. Mirhosseini N, Gray P L, Hoppes S, Tizard I, Shivaprasad H L, Payne S. Proventricular dilatation disease in cockatiels (*Nymphicus hollandicus*) after infection with a genotype 2 avian bornavirus. J Avian Med Surg. 2011; 25(3): 199–204. PMID: 22216720.
5. Philadelpho NA, Rubbenstroth D, Guimaraes MB, Piantino Ferreira AJ. Survey of bornaviruses in pet psittacines in Brazil reveals a novel parrot bornavirus. Vet Microbiol.2014;174(3–4):584–90. doi:10.1016/j.vetmic.2014.10.020PMID:25465670.
6. Raghav R, Taylor M, Delay J, Ojkic D, Pearl D L, Kistler A L, et al. Avian bornavirus is present in many tissues of psittacine birds with histopathologic evidence of proventricular dilatation disease. J Vet Diagn Invest. 2010; 22(4):495–508. PMID:20622218.
7. Rubbenstroth D, Rinder M, Kaspers B, Staeheli P. Efficient isolation of avian bornaviruses (ABV) from naturally infected psittacine birds and identification of a new ABV genotype from a salmon-crested cockatoo (*Cacatua moluccensis*) Vet Microbiol. 2012, 161 (1-2), 36-42
8. Rubbenstroth D, Schmidt V, Rinder M, Legler M, Twietmeyer S, Schwemmer P, et al. Phylogenetic Analysis Supports Horizontal Transmission as a Driving Force of the Spread of Avian Bornaviruses. PLoSONE. 2016; 11(8):e0160936. doi:10.1371/journal.pone.0160936
9. Weissenböck H, Bakonyi T, Sekulin K, Ehrensperger F, Doneley R J, Dürrwald R, et al. Avian bornaviruses in psittacine birds from Europe and Australia with proventricular dilatation disease. Emerg Infect Dis. 2009;15(9):1453–9. PMID:19788814. doi:10.3201/eid1509.090353
